# Monitoring and Maintaining the Freshness of Royal Jelly: A Review of Analytical Approaches and Preservation Technologies

**DOI:** 10.3390/foods14244300

**Published:** 2025-12-14

**Authors:** Yu Zhang, Jing Sun, Rui Chen, Lin Zhang, Xuan Ma, Jiangying Heng, Feng Wang, Xiaofeng Xue

**Affiliations:** 1 College of Biochemical Engineering, Beijing Union University, Beijing 100101, China; 2 State Key Laboratory of Resource Insects, Institute of Apiculture Research, Chinese Academy of Agricultural Sciences, Beijing 100193, China

**Keywords:** royal jelly, freshness evaluation, quality deterioration, preservation technologies

## Abstract

Royal jelly, a high-value natural product rich in bioactive compounds, is highly susceptible to quality deterioration during storage and processing. However, current quality standards rely predominantly on basic physicochemical parameters and measuring the content of 10-hydroxy-2-decenoic acid (10-HDA), which fail to capture the comprehensive and dynamic nature of its freshness. This significant knowledge gap hinders the accurate assessment, prediction, and control of royal jelly quality throughout its supply chain. To address this limitation, this review systematically elucidates the molecular mechanisms underlying the deterioration of royal jelly freshness, including key pathways such as protein denaturation, Maillard reactions, enzymatic inactivation, and lipid oxidation, and analyzes the combined effects of intrinsic and extrinsic factors on its quality stability. It highlights the potential applications of novel biochemical markers—including major royal jelly proteins (MRJPs), Maillard reaction products, enzymatic activity indicators, and energy metabolites—while comparing the advantages and limitations of traditional chromatographic techniques with modern rapid sensing and spectroscopic analysis methods. Regarding preservation, a critical yet inadequately summarized area, this review systematically evaluates the applicability and limitations of various approaches, including low-temperature storage, drying treatments, non-thermal sterilization, microencapsulation, and modified atmosphere packaging. Future directions for integrated quality control are outlined, providing a theoretical basis for holistic quality management of royal jelly.

## 1. Introduction

Royal jelly is a complex natural secretion produced by the hypopharyngeal and mandibular glands of worker honeybees (*Apis mellifera*), which are dedicated to nourishing the queen bee and larvae. It plays a decisive role in caste differentiation and ontogenetic development. This milky, viscous substance is rich in proteins, lipids (including the characteristic 10-hydroxy-2-decenoic acid, 10-HDA), carbohydrates, vitamins, and minerals [1]. Consequently, it exhibits a variety of bioactivities, including antioxidant, anti-inflammatory, immunomodulatory, and anti-aging effects [2]. These valuable properties render royal jelly a promising ingredient for broad applications in functional foods, dietary supplements, and pharmaceutical and cosmetic products.

China is the world’s largest producer and exporter of royal jelly [3], with its annual production accounting for over 90% of the global output [4], with the majority exported to Japan via cold-chain logistics to ensure bioactive integrity, particularly in the freeze-dried powder form, which is occupying a steadily increasing proportion of exports [5,6]. However, its high moisture content and nutrient-rich matrix make royal jelly highly susceptible to post-harvest quality deterioration. Factors such as temperature, storage duration, and oxygen exposure can induce protein denaturation and degradation, Maillard reactions, loss of enzymatic activity, and microbial proliferation. These changes adversely affect its sensory attributes (e.g., color, odor, and viscosity) and, more critically, cause the irreversible loss of key bioactive components. This ultimately undermines the product’s purported efficacy and significantly diminishes its commercial value.

The current quality standard system for royal jelly is primarily based on conventional physicochemical parameters. In China, the 10-HDA content is used as a core quality indicator, with a stipulated minimum threshold of 1.8% [7]. However, growing evidence indicates that 10-HDA possesses considerable chemical stability under commonly used storage conditions. Consequently, its concentration often remains unchanged even as the functional activity of royal jelly declines, rendering it a poor indicator for detecting early-stage quality deterioration. This limitation has prompted researchers to explore more representative indicators of freshness. In recent years, novel molecular markers—such as conformational changes in major royal jelly proteins (MRJPs), the accumulation of Maillard reaction products, the kinetics of enzymatic activity loss, and shifts in energy metabolite profiles—have emerged as more reliable proxies for royal jelly freshness [8].

Therefore, systematically elucidating the mechanisms affecting royal jelly freshness, scientifically identifying reliable indicators, and developing rapid and effective detection and preservation techniques have become key research priorities in this field. Although several deterioration markers have been reported individually, a systematic framework linking mechanistic pathways to integrated evaluation and preservation strategies remains underdeveloped. This review aims to comprehensively summarize the latest advances in royal jelly freshness research, with a focus on the underlying mechanisms of quality deterioration, the scientific basis and application potential of emerging evaluation markers, and a critical appraisal of the existing detection and preservation technologies, providing a theoretical reference for the improvement of quality control systems and the upgrading of the royal jelly industry.

## 2. Evaluation Indicators of Royal Jelly Freshness

**Figure 1:**
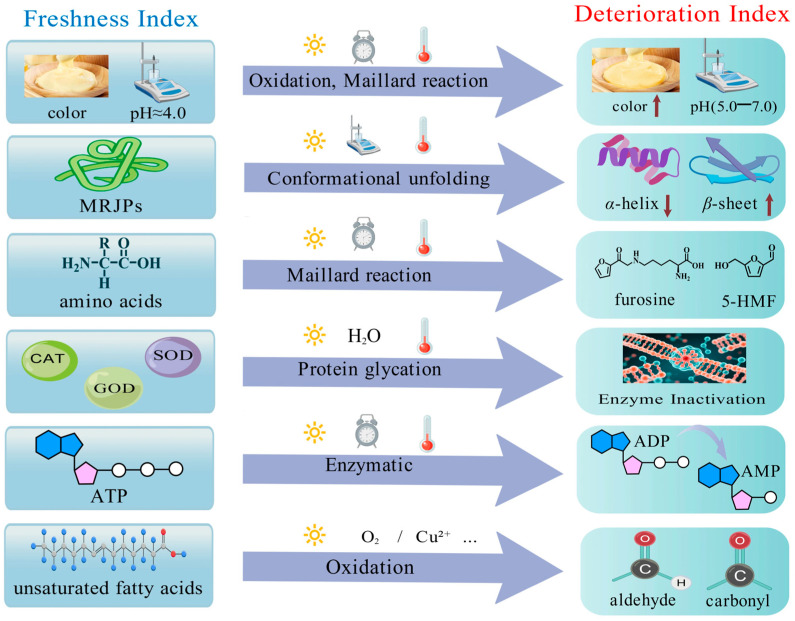
Evaluation indicators, mechanisms and factors influencing royal jelly freshness. Created with BioGPD.com.

The accurate evaluation of royal jelly freshness relies on a multidimensional analysis of indicators. The current research has progressed from traditional sensory and physicochemical assessments to a comprehensive system encompassing specific molecular markers, functional activities, and microbiological parameters, as shown in Figure 1.

### 2.1. Conventional Quality Parameters

#### 2.1.1. Sensory Characteristics

Sensory evaluation is the most traditional method for assessing the freshness of royal jelly, relying on intuitive judgments based on its color, aroma, taste, and texture. Fresh royal jelly typically appears as milky white or pale yellow, with a slight acidic taste and characteristic fragrance. During storage, the Maillard reaction and subsequent melanoidin formation produce high-molecular-weight, brown, water-insoluble nitrogenous compounds, thereby causing color darkening and increased viscosity, respectively. Concurrently, lipid oxidation is responsible for the development of rancid odors [9]. Although sensory evaluation offers the advantages of speed and does not require complex instruments, it is highly subjective and poorly reproducible. To address this, intelligent sensory technologies such as electronic tongues (e-tongues) and electronic noses (e-noses) have been introduced to achieve objective and quantitative assessments [10]. Li et al. [11] demonstrated that metal-oxide-semiconductor-based e-noses can accurately discriminate samples from different storage periods, while e-tongues can provide semi-quantitative predictions of acidity and bitterness changes.

#### 2.1.2. Physicochemical Parameters

Physicochemical parameters are currently the most widely used indicators for assessing the freshness of royal jelly, mainly including acidity, pH, volatile organic compounds (VOCs), and protein degradation products. Among these, acidity is particularly sensitive to storage time, showing a significant increase as the storage duration extends [12]. In addition, changes in the composition of VOCs can also effectively reveal the process of quality deterioration [13]. The advantage of physicochemical detection indicators lies in their objectivity, quantifiability, and traceability. However, individual physicochemical markers are easily influenced by raw material variations, collection season, and processing conditions, making it difficult to independently reflect overall freshness. Moreover, metabolomics studies have revealed significant fluctuations in amino acid profiles, organic acids, and certain aldehyde/ketone volatiles during storage, providing new avenues for identifying early freshness warning markers.

### 2.2. Biomolecular Indicators

#### 2.2.1. Major Royal Jelly Proteins

The stability of MRJPs serves as a core molecular indicator for assessing royal jelly freshness. Since protein denaturation involves alterations in spatial structure, it cannot be directly observed through sensory evaluation, and thus requires corresponding technical methods for detection. Proteomic studies based on liquid chromatography–tandem mass spectrometry (LC-MS/MS) have shown that MRJP1, MRJP4, and MRJP5 are highly sensitive to temperature, with their levels decreasing significantly as storage temperature rises and storage duration lengthens, directly correlating with the loss of royal jelly’s biological functions [14,15]. Among them, MRJP1 undergoes a conformational transition from α-helix to β-sheet under thermal stress, leading to aggregation and precipitation, and its degradation products are considered as characteristic molecular markers of royal jelly aging [16]. In addition, MRJP degradation products are rich in residues such as lysine and glutamine, which readily participate in Maillard reactions and oxidative cross-linking, leading to color darkening, the loss of enzymatic activity, and off-flavor development. Compared with traditional physicochemical indicators, systematic changes in the MRJP secondary structure can serve as sensitive early signals of royal jelly freshness deterioration [17]. It is important to note that MRJP degradation kinetics can be influenced by bee lineage, diet, and processing methods, necessitating standardized detection protocols for reliable application.

#### 2.2.2. Maillard Reaction Products

The Maillard reaction is a primary chemical pathway responsible for browning and nutrient loss in royal jelly. Furosine content, as an early marker of this reaction, shows a stable increase in royal jelly with rising storage temperatures and prolonged storage time, and has been incorporated into international standards for royal jelly [18]. Its high sensitivity to thermal history provides quantitative evidence of quality deterioration, often before sensory or functional changes are detectable. However, furosine levels are influenced by matrix composition and processing conditions, and do not directly reflect protein conformational changes or alterations in biological activity [19]. Therefore, furosine should be used in conjunction with other functional indicators to establish a multidimensional system for assessing royal jelly freshness.

5-hydroxymethylfurfural (5-HMF) is a mid-to-late-stage product of the Maillard reaction and has been adopted by the Codex Alimentarius and the European Union as an indicator of royal jelly freshness [20]. Its concentration is extremely low in fresh royal jelly but undergoes an exponential increase during ambient storage, making it a sensitive marker for medium- to long-term storage at room temperature. Unlike relatively static markers like 10-HDA, the dynamic accumulation of 5-HMF allows for a more accurate reflection of the product’s actual storage history and thermal exposure. As its concentration can be artificially elevated during improper sample handling, it is recommended to store samples immediately at 4 °C for short-term preservation or at −20/−80 °C for long-term storage to ensure that analytical results accurately represent the original product quality. Meanwhile, Nε-carboxymethyl lysine (CML), a late-stage product of the Maillard reaction, exhibits exponential growth with extended storage time at room temperature, making it a potential indicator for assessing the freshness of royal jelly [21].

#### 2.2.3. Enzyme Activity

GOD and SOD are key bioactive enzymes in royal jelly, and both are highly sensitive to temperature fluctuations. Ali et al. [22] reported that GOD and other antioxidant enzyme activities decline significantly within weeks to months at room temperature or higher, while frozen storage markedly decelerates this activity loss. Dong et al. [23] further demonstrated that GOD activity can decrease by over 97% within 55 days at 27–30 °C. Therefore, enzymatic activity serves as an excellent indicator for assessing royal jelly freshness, especially under short-term storage or cold chain interruptions. The key advantage of using enzyme activity as a marker lies in its direct correlation with antibacterial and antioxidant functions and its sensitivity to short-term temperature fluctuations. However, enzyme activity is easily influenced by sample dilution, protein degradation, and endogenous inhibitors; therefore, the reliance on a single threshold means that results are prone to misinterpretation [24]. Thus, it is advisable to use enzyme activity in combination with other indicators to establish a more reliable and comprehensive freshness evaluation system.

#### 2.2.4. Energy Metabolites

Adenosine phosphate compounds in royal jelly, such as adenosine triphosphate (ATP), adenosine diphosphate (ADP), and adenosine monophosphate (AMP), directly reflect its post-harvest energy metabolism status. After removal from the bee, endogenous enzymatic systems remain active, driving the sequential degradation of ATP into ADP, AMP, and other products [25]. This degradation process, governed by both temperature and time, provides a kinetic profile that can quantitatively indicate the metabolic decay rate of royal jelly and the integrity of the cold chain [26]. Xue et al. [27] developed an accelerated solvent extraction coupled with online purification and the high-performance liquid chromatography (HPLC) method, significantly improving the extraction efficiency and analytical throughput of these compounds in complex matrices. Energy metabolites can capture early quality deterioration signals at the biochemical level. However, their detection still faces challenges such as complex sample preparation and sensitivity to storage media and pH. Future efforts should focus on standardizing extraction procedures and integrating them with non-destructive detection techniques to promote industrial applications.

## 3. Mechanisms and Factors Influencing Deterioration of Royal Jelly Freshness

The post-harvest deterioration of royal jelly is a dynamic process of molecular-level changes during storage, influenced by a variety of intrinsic and extrinsic factors. The systematic elucidation of the underlying degradation mechanisms and the impact of external factors is therefore essential for establishing a scientific basis for precise quality control. This section delves into these key deterioration pathways and their driving factors to provide a mechanistic understanding of freshness loss.

### 3.1. Mechanisms of Deterioration of Royal Jelly Freshness

The quality deterioration of royal jelly during storage primarily stems from its inherent physicochemical properties, such as high moisture content, highly concentrated nutrients, and the structural lability of various active components. These characteristics render it susceptible to a series of molecular-level changes during storage. Specifically, proteins may undergo conformational disruption and degradation; Maillard reaction products between sugars and amino acids gradually accumulate, affecting color and flavor; endogenous enzymes of significant functionality progressively lose activity; and some lipid components are prone to oxidation, as shown in Figure 1. These processes often occur concurrently and exhibit synergistic effects, ultimately leading to a decline in the sensory quality, diminished nutritional functionality, and reduced bioactivity of royal jelly. Moreover, such deterioration is typically irreversible.

#### 3.1.1. Denaturation and Degradation of MRJPs

MRJPs, which constitute the core architectural proteins of royal jelly, are highly susceptible to denaturation under environmental stressors such as elevated temperature, pH fluctuations, and oxidative stress. As demonstrated by Qiao et al. [21], MRJPs undergo partial conformational unfolding during storage, characterized by a decrease in α-helix and β-turn content and a concomitant increase in β-sheet structures. This unfolding is accompanied by alterations in tertiary structure, evidenced by red shifts in intrinsic fluorescence emission spectra and a decrease in fluorescence intensity, which indicate the exposure of hydrophobic residues. The denatured proteins subsequently tend to aggregate, leading to a significant loss of solubility and antioxidant capacity [28]. Under prolonged or severe storage conditions, this denaturation and aggregation can progress to irreversible degradation. Proteomic studies using techniques such as liquid chromatography–tandem mass spectrometry (LC-MS/MS) have confirmed the breakdown of specific MRJPs (including MRJP1, MRJP2, MRJP3, and MRJP5) into fragments during storage, providing direct molecular evidence of protein degradation [14]. Consequently, the denaturation, aggregation, and eventual degradation of MRJPs serve as a primary indicator and direct cause of freshness loss [29].

#### 3.1.2. Maillard Reaction

The Maillard reaction represents a key non-enzymatic chemical pathway responsible for the browning and concomitant nutritional degradation of royal jelly. This reaction occurs between the amino groups of proteins or free amino acids and the carbonyl groups of reducing sugars, culminating in the formation of a series of advanced glycation end-products [30,31]. The rate of the Maillard reaction is highly temperature-dependent. It proceeds slowly at room temperature, increasing markedly with elevated temperatures. For instance, Chen et al. [10] reported a significantly lower content of reducing sugars in royal jelly stored at room temperature or under refrigeration compared to frozen samples. Similarly, Marconi et al. [32] observed a marked increase in furosine content in royal jelly following ten months of storage at room temperature. As an early marker of the Maillard reaction, furosine is widely utilized to evaluate the severity of heat treatment and quality deterioration during storage [12].

5-HMF is a mid-to-late-stage product of the Maillard reaction. Ciulu et al. [20] monitored royal jelly stored at −18, 4, and 25 °C for nine months using a reversed-phase high-performance liquid chromatography (RP-HPLC) method. They found that while the 5-HMF concentration in all samples stored at 4 and −18 °C was below the detection limit, its concentration in samples stored at 25 °C increased exponentially with storage time. Meanwhile, CML is one of the markers for advanced glycation end-products in the Maillard reaction. Qiao et al. [21] determined the CML content in royal jelly samples stored at room temperature (approximately 25 °C) for 1 to 6 months. The results showed that the CML content increased by approximately sixfold during the six-month room temperature storage period, indicating that the levels of both 5-HMF and CML in royal jelly are related to its freshness.

However, the formation of Maillard reaction products is influenced by factors such as the sugar content of the matrix, product formulation, and processing methods like drying. Therefore, a comprehensive assessment of freshness typically requires the integration of furosine, 5-HMF, and CML data with other indicators, such as protein degradation profiles, enzymatic activity, and sensory evaluation [33].

#### 3.1.3. Enzymatic Inactivation

Endogenous bioactive enzymes, including glucose oxidase (GOD), superoxide dismutase (SOD), and catalase (CAT), constitute a vital natural defense system in royal jelly, conferring intrinsic antimicrobial and antioxidant activities. However, these enzymes are highly labile and susceptible to inactivation under stressors such as high temperature, high humidity, or light exposure, thereby compromising this endogenous defense system [34]. Li et al. [29] observed that the storage of royal jelly at room temperature for one year led to an almost complete loss of GOD and CAT activities. Notably, enzymatic activity declines more substantially under refrigeration than under frozen storage. This inactivation is primarily attributed to temperature-induced conformational unfolding of the enzyme proteins, which exposes their catalytic sites to oxidative damage and potential proteolytic cleavage. Furthermore, hydrogen peroxide generated by active glucose oxidase may lead to the production of reactive oxygen species through enzymatic or metal-catalyzed pathways. These reactive species can subsequently oxidize critical amino acid residues within the enzyme structure, contributing to irreversible activity loss, as supported by studies on enzyme stability [35]. Cold and frozen storage mitigates enzyme inactivation by restricting molecular motion and reducing the generation of hydrogen peroxide and its derived free radicals, underscoring the critical importance of maintaining a low-temperature chain to preserve enzymatic function [36].

#### 3.1.4. Lipid Oxidation

Royal jelly is rich in unsaturated fatty acids. Its characteristic component, 10-HDA, is particularly susceptible to autoxidation upon exposure to light, oxygen, and metalion catalysts, leading to the formation of secondary oxidation products including hydroperoxides, aldehydes, and ketones. The autoxidation process is initiated by the reaction of lipid radicals with molecular oxygen (O_2_), forming peroxyl radicals that subsequently abstract hydrogen to generate unstable lipid hydroperoxides. The decomposition of these hydroperoxides yields reactive short-chain aldehydes and carbonyl compounds. These secondary products can adduct to or cross-link with amino acid residues in proteins, causing functional site damage, protein aggregation, and a consequent decline in biological activity [37]. Chen et al. [10] analyzed royal jelly stored at different temperatures using headspace solid-phase micro-extraction gas chromatography–mass spectrometry (HS-SPME-GC/MS). They found that the levels of lipid oxidation products increased significantly over time, concurrent with a rise in fatty acid degradation products, indicating an acceleration of lipid peroxidation. This oxidative process not only generates undesirable rancid odors but also disrupts the structural integrity of the lipid matrix, thereby significantly impairing the antioxidant and antimicrobial properties of royal jelly [34].

### 3.2. Influencing Factors of Royal Jelly Freshness

The deterioration of royal jelly quality during storage stems from a complex interplay between its intrinsic chemical composition and external environmental conditions [38]. As inferred from the aforementioned molecular mechanisms, the rate of freshness loss is co-modulated by intrinsic, extrinsic, and biological factors. Internal factors define the inherent susceptibility and potential pathways for deterioration, whereas external factors act as modulators that directly influence the kinetics of these degradation reactions and microbial growth. Biological factors operate upstream, influencing the initial compositional profile of royal jelly and thereby predetermining its baseline stability for subsequent storage.

#### 3.2.1. Intrinsic Factors

Internal factors (water activity, pH, viscosity, acidity, color) constitute the fundamental material basis for the quality stability of royal jelly and are defined by its intrinsic physicocl properties.

High moisture content is a central intrinsic condition driving multiple deterioration reactions. With a moisture content reaching 60–70%, royal jelly provides a suitable medium for its endogenous enzymes while also creating a favorable environment for the colonization and proliferation of external microorganisms [39]. This high water activity significantly accelerates enzymatic hydrolysis, Maillard reactions, and microbial metabolism, resulting in increased viscosity, elevated acidity, and the deterioration of flavor.

The initial stability of bioactive components is critical in determining the functional lifespan of royal jelly. Royal jelly is naturally acidic [4], and this acidic environment is crucial for the stability of MRJPs within it. Mureşan et al. [40] found that when MRJPs transition from their synthesis environment (pH ≈ 7.0) to the secretion environment of royal jelly (pH ≈ 4.0), both their protein conformation and thermal stability are optimized. Furthermore, pH affects the texture and stability of royal jelly. Buttstedt et al. [41] discovered that when the pH shifts from acidic (approximately 4.0) to neutral (e.g., pH 5–7), royal jelly changes from a gel-like state to a thinner liquid, which may adversely affect its storage stability as well as sensory properties (color, texture). Therefore, pH is extremely important for maintaining the freshness, physicochemical quality, and biological activity of royal jelly.

#### 3.2.2. Extrinsic Factors

External factors function as primary levers for quality regulation. The main way to affect the preservation effect of the product is by altering the rate of the degradation reaction, and this is the most controllable variable in the production and supply chain process.

Temperature is the primary driving factor for all degradation reactions. According to chemical reaction kinetics, elevated temperatures significantly accelerate protein denaturation, Maillard reactions, lipid oxidation, and enzyme inactivation [42]. MRJPs and bioactive macromolecules such as GOD have native conformations that are highly sensitive to fluctuations in temperature and pH [34]. The evidence indicates that MRJP1 is more prone to irreversible denaturation under thermal stress compared to the chemically stable 10-HDA, positioning it as a more promising early warning indicator of freshness loss [43]. Although the initial content of the characteristic fatty acid 10-HDA is influenced by genetic and dietary factors [44], its concentration only changes minimally during storage. This stability renders it insufficient for monitoring the early stages of functional activity decline in the product. Consequently, storage at room temperature can lead to a significant deterioration of the sensory and functional properties of royal jelly within just a few weeks [45]. In contrast, low temperatures, particularly freezing, maximally suppress these reaction rates and effectively extend shelf life.

Storage duration is a critical factor leading to the irreversible deterioration of the freshness and functional quality of royal jelly. This deterioration is a complex and dynamic process involving multiple components and pathways. MRJPs undergo denaturation, aggregation, and degradation, resulting in a significant reduction in their water solubility and antibacterial activity [14,17]. Prolonged storage also triggers the Maillard reaction and lipid oxidation, causing undesirable changes in the product’s color and texture, as well as the development of rancid odors [20]. The duration of storage ultimately dictates the cumulative extent of degradation product formation.

Oxygen and light are the primary triggers of oxidative rancidity and photochemical degradation. Molecular oxygen is an essential reactant in the radical chain reactions of lipid autoxidation, directly leading to the degradation of unsaturated fatty acids, including 10-HDA, and the generation of volatile off-flavor compounds [46]. Exposure to light, particularly ultraviolet radiation, can directly initiate lipid-free radical reactions and also catalyze Maillard browning. These photochemical processes collectively contribute to color darkening and the development of undesirable flavors.

#### 3.2.3. Biotic Factors

Biological factors predominantly exert their influence during the secretion phase of royal jelly. They regulate its initial biochemical composition and thereby establish the fundamental conditions that predetermine its subsequent storage stability.

The initial microbial load is a pivotal factor governing both the shelf life and safety of royal jelly. Hygiene standards during collection, the health status of the bee colony, and operational practices directly dictate the initial total viable count. Elevated microbial loads act as biological catalysts, not only promoting fermentation and spoilage but also accelerating protein degradation and the formation of off-flavors, thereby exacerbating the overall rate of quality deterioration [13].

Colony nutrition and gene expression are upstream factors influencing the initial composition of royal jelly. The forage source, particularly the botanical origin of pollen, influences the development of the hypopharyngeal glands in nurse bees and modulates the expression of genes encoding royal jelly proteins, such as MRJP1. Research has demonstrated that while the 10-HDA content may remain comparable in royal jelly produced from different pollen diets, the protein profiles and the activities of key enzymes like GOD can exhibit significant variations [47]. These findings indicate that nutritional input directly modulates honeybee physiology at a molecular level, leading to alterations in the biochemical composition of royal jelly that precondition its stability during subsequent storage.

## 4. Analytical Techniques for Royal Jelly Freshness

The accurate assessment of royal jelly freshness relies on reliable analytical techniques. Currently, the field is transitioning from offline, single-point analyses dependent on laboratory precision instruments toward rapid, non-destructive, multi-indicator methods, which are suitable for on-site industrial application. This evolution is forming an integrated system that combines laboratory-grade quantification with real-time, on-site screening, as shown in Table 1.

### 4.1. Laboratory Quantitative Analysis Techniques

#### 4.1.1. Chromatographic and Mass Spectrometric Techniques

HPLC is extensively employed for the quantitative analysis of 10-HDA [48], furosine, and 5-HMF, enabling the monitoring of compositional changes in royal jelly during storage. Wu et al. [49] developed a rapid ultra-high-performance liquid chromatography (UHPLC) method for separating and quantifying 26 amino acids in royal jelly. Their analysis of free and total amino acid profiles in samples stored under different conditions revealed significant, continuous decreases in total methionine and free glutamine levels, suggesting their potential as quality control parameters.

LC-MS/MS serves as a cornerstone technique for quantifying MRJPs, Maillard reaction products, and ATP metabolites. It enables the highly sensitive detection of characteristic peptide profiles derived from the denaturation and hydrolysis of MRJPs, providing molecular-level evidence for assessing the loss of bioactivity during storage or processing. The technique also enables precise quantification of both early-stage Maillard reaction products, such as furosine, and mid-to-late-stage compounds like 5-HMF [50], achieving detection limits substantially lower than those of conventional HPLC [51]. Despite its exceptional precision and capability for simultaneous multi-component analysis, the method remains constrained by high instrument costs and operational complexity, making it applicable for laboratory-based quantitative analysis.

Ultra-performance liquid chromatography–tandem mass spectrometry (UPLC-MS/MS), nanoflow liquid chromatography–tandem mass spectrometry (nanoLC-MS/MS), and matrix-assisted laser desorption ionization–time-of-flight mass spectrometry (MALDI-TOF MS) are applied for the detection of MRJPs in royal jelly via proteomic methodologies. Lin et al. [52] employed UPLC-MS/MS for the quantitative analysis of royal jelly. The results indicated that the content of MRJP1 in fresh royal jelly ranged from approximately 41.96 to 55.01 mg/g. This method can be applied to quantify MRJP1 in royal jelly and assess its freshness. Li et al. [29] conducted a systematic analysis of changes in the protein composition of royal jelly under different storage conditions and durations using nanoLC-MS/MS and MALDI-TOF/TOF-TOF proteomic approaches. The study revealed that as storage temperature increased or duration extended, significant alterations occurred in the types, abundance, and aggregation states of MRJPs (particularly MRJP1-3) and enzymes such as glucose oxidase. Notably, MRJP5 was even found to disappear after long-term room temperature storage, suggesting its potential as a sensitive marker for freshness assessment.

#### 4.1.2. Immunoassay Techniques

The enzyme-linked immunosorbent assay (ELISA), based on specific antigen–antibody interactions, enables the targeted quantification of proteins such as MRJP1, providing a direct measure of royal jelly freshness. This approach shows significant potential for development into commercial quantitative test kits. Shen et al. [53] developed a polyclonal antibody against MRJP1-specific peptides and established an indirect ELISA that demonstrated progressively decreasing MRJP1 content with extended storage duration. It was found that the freshness of royal jelly can be characterized by monitoring the content of MRJP1. In addition, the results obtained by the indirect ELISA method used by Yamaguchi et al. [54] were consistent with those obtained by the HPLC or gel filtration methods. This confirmed the accuracy and practicability of this method.

In practical applications, ELISA provides more direct and sensitive protein quantification compared to HPLC, which is typically reserved for small molecules like 10-HDA, amino acids, and 5-HMF. This makes ELISA particularly suitable for assessing protein degradation. The future development of standardized ELISA-based kits could enable rapid, routine freshness assessment throughout production, distribution, and quality control facilities.

### 4.2. Rapid and Non-Destructive Screening Techniques

#### 4.2.1. Spectroscopic Techniques

FTIR and near-infrared (NIR) spectroscopy enable rapid, non-destructive prediction of multiple quality parameters in royal jelly, including moisture, protein, and 10-HDA content, providing insights into storage history and degradation degree. Tarantilis et al. [55] utilized FTIR spectroscopy to demonstrate that royal jelly proteins remain stable for 3 days at room temperature, 7 weeks at 4 °C, and 21 weeks at −20 °C, showing that it can monitor the degradation of royal jelly proteins. Furthermore, data fusion of FTIR and NIR spectra significantly improves the prediction accuracy for 10-HDA and protein content [56]. These spectroscopic techniques offer rapid analysis, non-destructive sampling, and potential for online monitoring, making them ideally suited for high-throughput screening in production and storage environments as potential alternatives to conventional chemical analyses.

#### 4.2.2. Sensor-Based Techniques

E-noses and e-tongues represent emerging non-destructive tools for royal jelly freshness evaluation through detection of VOCs and comprehensive flavor profiling. Their core mechanism lies in the fact that during the storage process, VOCs and small molecules related to flavor will undergo chemical reactions, such as oxidation, dehydrogenation, or esterification reactions. These reactions will cause measurable changes in the sensor output, including changes in conductivity or potential patterns. Chen et al. [10] employed HS-SPME-GC/MS combined with e-nose and e-tongue analyses to monitor royal jelly stored at 25 °C for 21 days, observing a significant increase in VOCs that correlated with sensory-detected off-flavors and bitterness. Combining this with metabolomics methods to verify the characteristics of volatile components and developing a comprehensive electronic fingerprint database can provide intelligent support for rapid sensory-based quality control.

#### 4.2.3. Quick Detection Techniques

Immunochromatographic test strips, based on specific antibodies, can detect MRJPs and enzymatic activities within 10–15 min, enabling the rapid assessment of royal jelly freshness. Immunochromatographic test strips achieve qualitative or semi-quantitative detection of MRJP4 through specific binding between antibodies and MRJP4 [57]. Hu et al. [17] developed a colloidal gold immunochromatographic test strip for the detection of MRJP4 in royal jelly. This method offers the advantages of simple operation and short detection time, making it suitable for rapid on-site freshness assessment.

Colorimetric sensors, based on chemical-reaction-induced color changes, provide another rapid assessment approach for indicators including 10-HDA and furosine. Compared to HPLC with its complex sample preparation, colorimetric sensors offer advantages of lower cost and faster operation. Zheng et al. [58] established a simple colorimetric method using 37.5% HCl that completes freshness assessment within 6 min, suitable for real-time quality determination during commercial transactions. This method capitalizes on the significant decrease in 10-HDA content during storage, particularly under elevated temperatures [59]. Su et al. [60] developed a colorimetric method based on the 10-HDA–Ag^+^ coordination, which effectively inhibits 3,3’,5,5’-tetramethylbenzidine oxidation. This inhibition induces a distinct blue-to-colorless transition, enabling accurate 10-HDA quantitation and demonstrating strong potential for on-site freshness screening of royal jelly.

Overall, detection technologies for royal jelly freshness are gradually shifting from offline and time-consuming methods to online, rapid, and non-destructive approaches. The focus is evolving from single-parameter evaluation to multi-parameter integration, and from laboratory-based analysis toward portable and intelligent detection systems.

**Table 1:** Comparison of analytical techniques for royal jelly freshness.

Technique	Detectable Indicators	Advantages	Limitations	Typical Applications	Refs.
HPLC	10-HDA, furosine, 5-HMF, amino acids	High quantitative accuracy and reproducibility; multi-component analysis	Time-consuming sample preparation; not suitable for on-site use	Storage stability monitoring; quality control during processing	[49]
LC-MS/MS	MRJPs, 10-HDA, maillard reaction products, amino acids	High sensitivity and selectivity; provides structural information	High equipment cost; complex operation; lengthy procedures	Laboratory quantification; standardized quality control	[49,51]
UPLC-MS/MS	MRJPs	Suitable for the quantification of complex mixtures; offers accurate quantification and good reproducibility	Typically targets only a few marker proteins and may overlook low-abundance proteins	Quantitative freshness assessment	[52]
nanoLC-MS/MS	Proteins, enzymes	Capable of detecting low-abundance proteins; well-suited for revealing proteomic changes induced by storage	The sample pretreatment process is complex; high-abundance proteins may mask low-abundance components	Studying the mechanisms of protein degradation, aggregation, and structural alterations	[29]
MALDI-TOF MS	Proteins, protein fingerprints	The method is rapid and high-throughput, requiring no complex separation steps	It is difficult to perform absolute quantification; the establishment of databases or spectral libraries demands high standards	Adulteration identification and rapid screening	[29]
ELISA	MRJP1, MRJP4	High throughput, rapid, and highly specific; kit development potential	Limited to known protein targets; indirect degradation assessment	On-site protein quantification; production line monitoring	[53,54]
FTIR/NIR	Moisture, proteins, 10-HDA	Rapid, non-destructive; high-throughput multi-parameter analysis	Requires model calibration; matrix interference possible	Online production monitoring; rapid quality screening	[55]
E-Nose/Tongue	VOCs, flavor profiles	Rapid, non-destructive; captures overall flavor fingerprint	Susceptible to environmental conditions; needs calibration	Storage duration assessment; batch consistency checking	[10]
Immunochromatographic Test Strip	MRJPs, specific enzyme activities	Rapid operation; suitable for field use	Semi-quantitative; target-specific; requires antibody development	Preliminary on-site screening; production line testing	[17,57]
Colorimetric Sensor	10-HDA, furosine	Low cost, simple operation, fast results	Moderate sensitivity; matrix interference	Raw material screening; semi-finished product testing	[58,59]

Note:  HPLC: high-performance liquid chromatography; LC-MS/MS: liquid chromatography–tandem mass spectrometry; UPLC-MS/MS: ultra-performance liquid chromatography–tandem mass spectrometry; nanoLC-MS/MS: nanoflow liquid chromatography–tandem mass spectrometry; MALDI-TOF MS: matrix-assisted laser desorption ionization–time-of-flight mass spectrometry; ELISA: enzyme-linked immunosorbent assay; FTIR: Fourier transform infrared spectroscopy; NIR: near-infrared; e-tongues: electronic tongues; e-noses: electronic noses.

## 5. Preservation Technologies for Royal Jelly Freshness

To delay royal jelly quality deterioration during storage, various physical and physicochemical preservation techniques have been developed and applied, as shown in Table 2. These methods primarily function by inhibiting microbial growth, reducing the rate of chemical reactions, and protecting the structural stability of functional components, thereby extending the product’s shelf life.

### 5.1. Physical Preservation Techniques

#### 5.1.1. Low-Temperature Storage Method

Low-temperature storage is the most fundamental and widely applied technique for maintaining the freshness of royal jelly. Freezing (typically at −18 °C) maximally suppresses enzyme activity, delays protein denaturation and lipid oxidation, and thereby better preserves its antioxidant capacity and bioactivity [10]. Research confirms that freezing conditions effectively minimize the aggregation and inactivation of MRJPs, substantially prolonging the duration of safe storage [61]. The effectiveness of this technique highly depends on a stable and uninterrupted cold chain, as repeated freeze–thaw cycles can induce irreversible protein denaturation. Therefore, royal jelly should be frozen immediately after harvest.

#### 5.1.2. Freeze-Drying Processing Method

Freeze-drying sublimates the water in royal jelly under low-temperature vacuum conditions, reducing its water activity to extremely low levels and thereby fundamentally inhibiting most chemical and microbial degradation reactions. This process maximally preserves heat-sensitive bioactive components, such as 10-HDA, proteins, and polyphenolic compounds [9]. Studies have shown that freeze-dried royal jelly powder at the nano- or micro-scale exhibits storage stability superior even to that of fresh samples, and that it can maintain its antioxidant and antibacterial activities for extended periods at room temperature, greatly facilitating storage and transportation [9].

#### 5.1.3. Non-Thermal Sterilization Method

High-pressure processing and moderate irradiation can effectively inactivate microorganisms at relatively low temperatures, offering unique advantages for preserving the natural composition and bioactivity of royal jelly. Pan et al. [62] demonstrated that high-pressure processing modifies protein tertiary and quaternary structures while preserving their secondary structures, effectively disrupting MRJP fibrillar aggregation, enhancing protein–water interactions, and improving solubility. However, an excessively high pressure may induce aggregation of protein subunits and irreversible denaturation [63]. Irradiation serves as a complementary technique to eliminate surface and internal microbial contamination. Appropriate doses of gamma or electron-beam irradiation can significantly extend shelf life, though precise dose control is essential to avoid free-radical-mediated damage to bioactive components [64]. The safety and functional efficacy of irradiated royal jelly require validation through metabolomic and bioactivity analyses [65].

### 5.2. Integrated Physico-Chemical Strategies

#### 5.2.1. Modified Atmosphere Packaging

Packaging and storage atmosphere serve as effective physical barriers against environmental stress. Modified atmosphere packaging preserves quality by altering the internal gas composition. The oxygen and moisture barrier properties, as well as the light-blocking ability, of packaging materials are critical. Vacuum and nitrogen-flushed packaging effectively reduce the oxygen partial pressure in the headspace by removing or displacing oxygen, thereby fundamentally slowing the kinetics of oxidative reactions [66,67] and protecting 10-HDA and flavor compounds [68,69]. This approach has been proven to be an effective strategy for maintaining the long-term storage stability of royal jelly.

Emerging nanomaterial-based packaging, such as bio-based films incorporated with nano-antimicrobial or antioxidant agents, can continuously exert their effects at the packaging interface, providing additional protection under ambient or mild cold-chain conditions [70]. The safety of such nanocomposites is crucial, and their component migration behavior must be strictly evaluated before application to ensure compliance with food contact material regulations.

#### 5.2.2. Microencapsulation

Microencapsulation technology entraps active royal jelly components within micro- or nano-scale matrices using wall materials such as maltodextrin, gum arabic, or chitosan, creating a physical barrier against environmental factors [71]. Processing techniques like spray-drying yield free-flowing powdered products with markedly enhanced stability at room temperature [72]. The choice of wall materials and processing parameters directly affects the encapsulation efficiency and the retention of bioactivity [73]. More advanced carrier systems, including alginate–pectin composite gels or protein–polysaccharide nanoparticles, not only provide exceptional storage stability [74] but also enable controlled release and improved bioavailability, opening new avenues for high-value royal jelly product development [75].

#### 5.2.3. Additive Protection

Incorporating protective additives during freeze-drying or microencapsulation effectively prevents protein denaturation throughout dehydration. The mechanism involves forming a high-glass-transition matrix and increasing the system’s glass transition temperature, thereby restricting molecular mobility and chemical reaction rates. This suppresses protein folding or aggregation and lipid oxidation during drying and storage. For instance, adding suitable protectants such as trehalose during freeze-drying can reduce protein conformational changes and enhance storage stability [76]. In practical applications, the formulation design must balance protection efficiency with sensory and processing properties.

**Table 2:** Comparison of preservation for royal jelly freshness.

Technology	Key Parameters	Quality Impact	Applications and Limitations	Refs.
Low-Temperature Storage	−18 °C freezing; stable cold chain required	Preserves MRJPs and 10-HDA; prevents enzymatic degradation	Universal application; cold-chain dependency critical	[34,77]
Freeze-Drying Processing	Low-temperature vacuum; high equipment cost	Maintains bioactivity; enables room-temperature storage	High-value products; energy-intensive process	[9,78]
Non-Thermal Sterilization Technologies	100–200 MPa; specialized equipment	Depolymerizes MRJPs; inhibits microbial growth	Medium–large scale; pressure optimization required	[52,62]
Ionizing Irradiation	Controlled dosage; regulated facilities	Reduces microbial load; potential antioxidant loss	Large-scale sterilization; regulatory compliance needed	[64,79]
Modified Atmosphere Packaging	Oxygen exclusion; barrier materials	Prevents oxidation; preserves flavor compounds	Multi-scale use; material selection crucial	[68,80]
Microencapsulation	Wall materials; spray-drying parameters	Protects bioactives; masks undesirable odors	Functional foods; formula optimization essential	[71,72]
Protective Additives	Glass transition modulation; low–medium cost	Stabilizes proteins; enhances storage stability	Wide applicability; sensory balance required	[76,81]

## 6. Conclusions and Prospects

This review synthesizes evidence that the deterioration of royal jelly freshness occurs through four interconnected physicochemical and biochemical pathways: protein denaturation and degradation of MRJPs, which impairs solubility and bioactivity; the Maillard reaction, progressing from early markers such as furosine to advanced products including 5-HMF and CML, leading to browning and nutrient loss; enzymatic inactivation, notably of glucose oxidase, compromising natural preservation systems; and lipid oxidation, which generates rancidity and degrades key components such as 10-HDA. Consequently, the quality evaluation paradigm is shifting from reliance on static indicators toward a multi-marker framework that utilizes these pathway-specific biomarkers for more accurate and dynamic assessment.

A variety of rapid screening technologies have emerged to address the need for practical, on-site monitoring. These include ELISA kits targeting MRJPs, which offer high specificity and potential for field deployment; FTIR/NIR spectroscopy for the non-destructive, multi-parameter detection of moisture, protein, and 10-HDA; electronic nose/tongue systems capable of capturing volatile flavor profiles associated with freshness loss; and low-cost colorimetric sensors for the rapid identification of indicators such as 10-HDA and furosine. These methods complement traditional laboratory-based chromatographic and mass spectrometric techniques, which remain essential for validation and standardization, but are less suited for rapid field use.

To bridge the gap between research and industry practice, a tiered and proactive quality control strategy is recommended. Initially, and fundamentally, harvested royal jelly should be immediately placed in light-proof, airtight containers and transferred to frozen storage to maximally inhibit all deterioration pathways and preserve its intrinsic freshness. For routine quality screening, producers can then adopt rapid, accessible methods to assess incoming batches. Subsequently, to enhance stability, reduce long-term storage and transport costs, and minimize quality loss, the conversion of raw royal jelly into freeze-dried powder is strongly advised. This process should be supported by optimized packaging, such as vacuum or nitrogen-flushed containers for both the raw material and the final powder, to prevent oxidation. All these on-site and processing measures should be periodically validated through standardized laboratory analysis of definitive biomarkers to ensure accuracy and maintain alignment with scientific standards.

Looking forward, several strategic directions emerge as critical for advancing the field. First, the development of integrated evaluation models that incorporate multivariate data from proteomic, metabolomic, and functional activity analyses represents a promising approach for comprehensive freshness assessment. Second, the convergence of artificial intelligence with spectroscopic and sensor technologies offers unprecedented opportunities for creating intelligent, portable detection platforms capable of real-time monitoring throughout the supply chain. Third, research should prioritize the development of sustainable preservation strategies, such as edible coatings and smart packaging systems, to reduce the dependence on energy-intensive methods. Additionally, the implementation of blockchain-enabled traceability systems coupled with international standardization efforts will be crucial for enhancing global quality governance and market competitiveness.

In conclusion, the transition from reductionist quality indicators to multidimensional evaluation frameworks, from laboratory-dependent analysis to field-deployable monitoring, and from conventional preservation to intelligent stabilization systems will define the future of royal jelly quality control. Through interdisciplinary integration and technological innovation, the field is poised to establish more scientifically grounded standards and operationally efficient practices, ultimately ensuring the preservation of royal jelly’s premium quality and biological efficacy while supporting the sustainable development of the apiculture industry.

## Data Availability

No new data were created or analyzed in this study. Data sharing is not applicable to this article.

## Abbreviations

10-HDA	10-hydroxy-2-decenoic acid
MRJPs	Major royal jelly proteins
FTIR	Fourier transform infrared spectroscopy
LC-MS	Liquid chromatography–mass spectrometry
LC-MS/MS	Liquid chromatography–tandem mass spectrometry
GOD	Glucose oxidase
SOD	Superoxide dismutase
CAT	Catalase
HS-SPME-GC/MS	Headspace solid-phase micro-extraction gas chromatography–mass spectrometry
e-tongues	Electronic tongues
e-noses	Electronic noses
VOCs	Volatile organic compounds
5-HMF	5-hydroxymethylfurfural
ATP	Adenosine triphosphate
ADP	Adenosine diphosphate
AMP	Adenosine monophosphate
HPLC	High-performance liquid chromatography
UHPLC	Ultra-high-performance liquid chromatography
ELISA	Enzyme-linked immunosorbent assay
RP-HPLC	Reversed-phase high-performance liquid chromatography
MALDI-TOF MS	Matrix-assisted laser desorption ionization–time-of-flight mass spectrometry
nanoLC-MS/MS	Nanoflow liquid chromatography–tandem mass spectrometry
UPLC-MS/MS	Ultra-performance liquid chromatography–tandem mass spectrometry
CML	Nε-carboxymethyl lysine
NIR	Near-infrared
